# A serious case of primary Raynaud's phenomenon in an infant

**DOI:** 10.1002/ccr3.1819

**Published:** 2018-09-17

**Authors:** Masaru Kobayashi, Ken‐ichi Takano, Junji Kamizono, Kotaro Ichikawa

**Affiliations:** ^1^ Pediatric Emergency Care Kitakyushu Municipal Yahata Hospital Kitakyushu, Fukuoka Japan

**Keywords:** fingertip necrosis, infant, primary Raynaud's phenomenon, vasodilators

## Abstract

We present a case of severe Raynaud phenomenon (RP) in an infant. The current strategy of RP treatment is incomplete; excluding secondary Raynaud phenomenon is vital as well. This case aims to help those with similar symptoms in the future by gathering data on cases.

## 1 INTRODUCTION

We present a case of primary Raynaud phenomenon (RP) over a period of 2 months in a 21‐month‐old boy. Despite prompt and intensive treatment, his fingertips turned black from the 8th day of hospitalization. However, the color showed improvement later, and he was discharged on the 45th day.

Raynaud's phenomenon was first reported in 1968. Primarily triggered by cold stimulation or stress, the condition causes reversible spasms of the peripheral arterioles, resulting in its characteristic triphasic changes.^1^ The triphasic changes are as follows. In the first phase, because of contraction of the peripheral arterioles, the fingertips become pale. In the second phase, because of vein stasis, cyanosis occurs. In the third phase, because of vasodilation, redness may develop, in addition to pulsating pain.

Severe ischemia causes significant pain and can lead to ulceration and necrosis if it persists. There are few case reports of primary RP in children and there is no clear approach to treating it; therefore, cumulative data from more cases are required to determine a treatment plan. Here, we present a case on the development of primary RP over a period of 21 months in an infant.

## 2 CASE PRESENTATION

A 21‐month‐old boy presented to our pediatric emergency care center in January 2017 with the chief complaint of cold extremities. His growth and developmental history were appropriate for his age, and he had no remarkable personal or family medical history.

**Figure 1:**
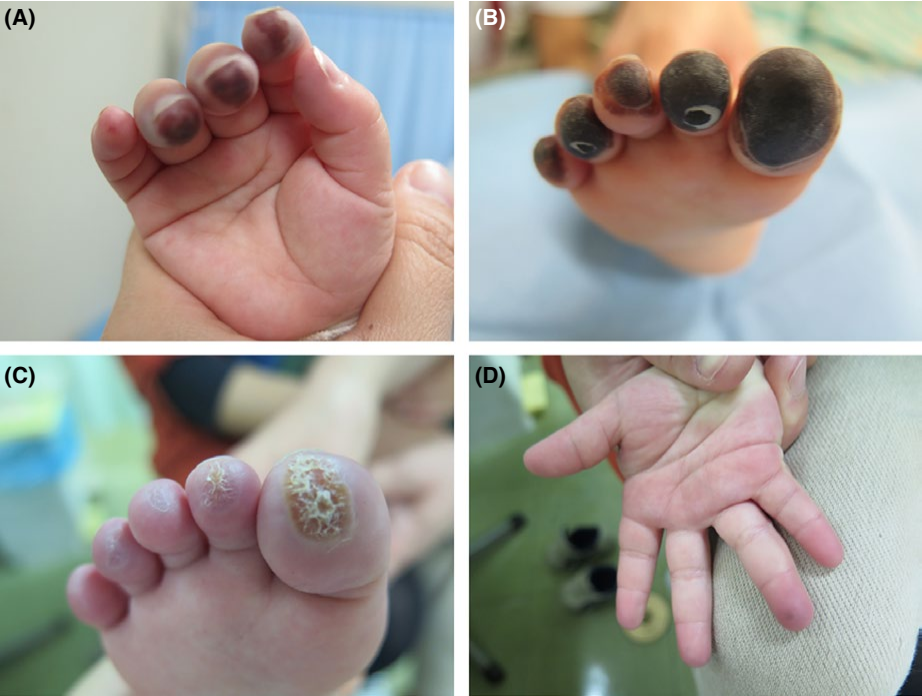
A, Fingertips of the right hand on the first day of hospitalization. Purple‐colored changes on the 2nd, 3rd, and 4th fingers are noted. B, The fingertips on the eighth day of hospitalization. Black‐colored changes in the 1st to 5th fingers are noted. C, Scab‐like lesions on the fingertips, index finger, and feet on day 18 of hospitalization. D, Finger cusp on day 40 of hospitalization. No change in color is noted

Transient fever and mild cough were noted 2 weeks before the consultation. He had been treated for frostbite for about 2 weeks by the previous doctor, but there had been no improvement. The child often played outdoors with bare hands at his nursery school. The patient subjectively complained of cold and painful fingers. Vital signs were normal, there was no cyanosis of the lips, and breathing sounds and heart sounds were normal. The abdomen was flat and soft, without hepatosplenomegaly and no palpable mass. His peripheral arterial pulse was detectable. (Figure 1A) However, his nailfold capillaries were difficult to observe. Laboratory analyses at the time of admission showed no leukocytosis, anemia, or platelet reduction. In addition, no increased liver enzymes or indications of renal dysfunction were observed, and C‐reactive protein and electrolyte levels were within the respective normal ranges.

Coagulation analyses showed a prolonged activated partial thromboplastin time (APTT) of 59.5 seconds, but the results normalized during hospitalization. The following parameters were normal: PT, 84%; fibrinogen, 169 mg/dL; and D‐dimer, 0.27 μ/mL. To diagnose mycoplasma infections, antibody titers (PA) were submitted at the time of admission, but they were <40 in both cases. Although lupus anticoagulant (LAC) positivity was noted only at the time of hospitalization, nuclear antibody levels, ds DNA antibody, anti‐RNP antibody, p‐ and c‐anti‐neutrophil cytoplasmic antibody levels, cold agglutinin reaction, rheumatoid factor, immunoglobulin levels, complement component levels, β2GP1 antibody levels, protein C levels, and protein S activity were within their respective normal ranges. Direct and indirect Coombs tests and cryoglobulin assessments also yielded negative findings. The electrocardiogram was normal, and no congenital heart disease was observed on echocardiography.

The contrast computed tomography (CT) and magnetic resonance angiography (MRA) of limbs were performed, but no obvious vasospasm was noted.

Although the application of a moisturizer and vasodilator on the extremities was initiated on the first day of hospitalization, the color of the fingertips symmetrically changed to black on the eighth day of hospitalization (Figure 1B). At the time of blood collection, no increase in inflammatory response was observed, and APTT, fibrinogen, and D‐dimer values were within the normal ranges.

A presumptive diagnosis of exacerbation of RP was made. The patient was treated with intravenous methylprednisolone for 3 days; in addition, oral administration of a calcium channel blocker and weekly administration of lipo‐prostaglandin E1 was initiated. Although the total amount of skin that had changed to black at the end of the limb gradually reduced, the skin that had already turned black did not revert to a normal tone.

On day 16, treatment with aspirin and traditional Chinese medicine was initiated. On day 18 of hospitalization, gradual peeling of the skin on the affected areas was observed (Figure 1C). Pathologic findings of the ends of the limbs showed an area of coagulation necrosis in the epidermis as well as dilated blood vessels, but no noticeable inflammation or vasculitis was observed in the surrounding area. Subsequently, the surface of the skin changed to a black‐gray crust and gradually peeled off. The skin surface below the skin that had peeled off was pinkish and normal. Thereafter, the color of the fingers improved, and the patient was discharged on the day 45 of hospitalization (Figure 1D).

At present, 1 year has passed without relapse of symptoms.

## 3 DISCUSSION

This case showed confirmed bilateral acrocyanosis.^2^ Initially, infant acrocyanosis, RP, reflex sympathetic dystrophy, hypoxemia, congenital heart disease, methemoglobinemia, and pheochromocytoma were all considered for the differential diagnosis.^3^ The possibility of RP was considered to be most probable, based on the change in the color tone at the end of the limb, the course of the symptoms, results of the blood analyses, and the echocardiogram. There were no obvious signs of infection or trauma associated with the appearance of the symptoms, and no vasculitis findings were identified on pathological examination. Furthermore, various collagen disease test results were in the normal ranges. Therefore, we diagnosed this disease as primary RP.

Based on the patient's history, the primary RP was assumed to be associated with cold stimulation. Of all patients with primary RP, approximately 13% are ultimately diagnosed with secondary RP, and studies have shown that exposure to cold is a major trigger for RP exacerbation.^4^ In laboratory evaluations, LAC showed positive findings only once (at admission) and β2 GP1 was absent. LAC was absent at the time of exacerbation of the symptoms, and the single positive result was considered to be related to fever before hospitalization.

The coagulation APTT test also showed a temporarily prolonged APTT concurrent with the positive LAC result, but the D‐dimer level did not increase. In addition, after 12 weeks, as the LAC and β2GP1 antibodies values were negative, the overall findings did not meet the diagnostic criteria for antiphospholipid syndrome.

The patient's condition improved with treatment over time. Insulation, protective measures against cold, Chinese medicine, steroid pulses, antiplatelet agents, vasodilators (calcium channel blockers), prostaglandin infusions, and antithrombotic therapies have been used for this disease with variable success rates; however, an optimal treatment for this disease has not yet been established.^5^, ^6^ As for the steroid pulses, since the possibility of secondary pediatric RP was considered at the time of use, it was based on the assumption of severe RP, per the treatment for mixed connective tissue disease.^7^

## 4 CONCLUSIONS

Temperature control is important for patients with primary RP. In addition, follow‐up assessment over time is essential when treating these patients. In the future, case sharing will be important to establish the optimal treatment protocol.

## CONFLICT OF INTEREST

None declared.

## AUTHOR CONTRIBUTIONS

MK: contributed to the design collection, analysis, and interpretation of the paper. KT, JK, and KI: were involved in the creation of a paper or critical review of important intellectual content. All authors have read and approved the final manuscript. This manuscript has not been published or presented elsewhere in part or in entirety and is not under consideration by another journal.

## References

[1] Saigal R , Kansal A , Mittal M , Singh Y , Ram H . Raynaud's phenomenon. J Assoc Physicians India. 2010;58:309‐313. 21117349

[2] Sharathkumar AA , Castillo‐Caro P . Primary Raynaud's phenomenon in an infant: a case report and review of literature. Pediatr Rheumatol Online J. 2011;9:16. 21767369 10.1186/1546-0096-9-16 PMC3162536

[3] DiMaio AM , Singh J . The infant with cyanosis in the emergency room. Pediatr Clin North Am. 2006;39:987‐1006. 10.1016/s0031-3955(16)38404-8 1523025

[4] Nigrovic PA , Fuhlbrigge RC , Sundel RP . Raynaud's phenomenon in children: a retrospective review of 123 patients. Pediatrics. 2003;111:715‐721. 12671102 10.1542/peds.111.4.715

[5] Vinjar B , Stewart M . Oral vasodilators for primary Raynaud's phenomenon. Cochrane Database Syst Rev. 2008;16:CD006687. 10.1002/14651858.CD006687.pub2 18425964

[6] Pope JE . Raynaud's phenomenon (primary). BMJ Clin Evid. 2013;2013:1119. PMC3794700 24112969

[7] Cassidy JT , Petty RE . Overlap syndrome/mixed connective tissue disease In Text Book of Pediatric Rheumatology, *5th edn* Philadelphia, PA: Elsevier Saunders; 2005:482‐486.

